# RETRACTION: Evaluating the Role of Shujing Tongdu Massage in Enhancing Wound Recovery and Alleviating Spinal Disease Symptoms: A Randomized Controlled Trial

**DOI:** 10.1111/iwj.70524

**Published:** 2025-04-18

## Abstract

**RETRACTION:** ZhangL. , XuW. , ZhangL. , CuiX. , and ChengF. , “Evaluating the Role of Shujing Tongdu Massage in Enhancing Wound Recovery and Alleviating Spinal Disease Symptoms: A Randomized Controlled Trial,” International Wound Journal 21, no. 1 (2024): e14633, 10.1111/iwj.14633.38272803 PMC10789518

The above article, published online on 15 January 2024, in Wiley Online Library (http://onlinelibrary.wiley.com/), has been retracted by agreement between the journal Editor in Chief, Professor Keith Harding; and John Wiley & Sons Ltd. Following an investigation by the publisher, all parties have concluded that this article was accepted solely on the basis of a compromised peer review process. The editors have therefore decided to retract the article. The authors did not respond to our notice regarding the retraction.

**RETRACTION:** L. Zhang , W. Xu , L. Zhang , X. Cui , and F. Cheng , “Evaluating the Role of Shujing Tongdu Massage in Enhancing Wound Recovery and Alleviating Spinal Disease Symptoms: A Randomized Controlled Trial,” International Wound Journal 21, no. 1 (2024): e14633, 10.1111/iwj.14633.38272803 PMC10789518

The above article, published online on 15 January 2024, in Wiley Online Library (http://onlinelibrary.wiley.com/), has been retracted by agreement between the journal Editor in Chief, Professor Keith Harding; and John Wiley & Sons Ltd. Following an investigation by the publisher, all parties have concluded that this article was accepted solely on the basis of a compromised peer review process. The editors have therefore decided to retract the article. The authors did not respond to our notice regarding the retraction.

